# The feature-specific propagation of orientation and direction adaptation from areas 17 to 21a in cats

**DOI:** 10.1038/s41598-017-00419-x

**Published:** 2017-03-24

**Authors:** Zhong Li, Jianjun Meng, Hongjian Li, Anqi Jin, Qijun Tang, Jianbin Zhu, Hongbo Yu

**Affiliations:** Vision Research Laboratory, School of Life Sciences, The State Key Laboratory of Medical Neurobiology, Collaborative Innovation Center for Brain Science, Fudan University, Shanghai, 200433 China

## Abstract

Adaptation plays a key role in visual information processing, and investigations on the adaptation across different visual regions will be helpful to understand how information is processed dynamically along the visual streams. Recent studies have found the enhanced adaptation effects in the early visual system (from LGN to V1) and the dorsal stream (from V1 to MT). However, it remains unclear how adaptation effect propagates along the form/orientation stream in the visual system. In this study, we compared the orientation and direction adaptation evoked by drifting gratings and stationary flashing gratings, as well as moving random dots, in areas 17 and 21a simultaneously of cats. Recorded by single-unit and intrinsic signal optical imaging, induced by both top-up and biased adaptation protocols, the orientation adaptation effect was greater in response decline and preferred orientation shifts in area 21a compared to area 17. However, for the direction adaptation, no difference was observed between these two areas. These results suggest the feature-specific propagation of the adaptation effect along the visual stream.

## Introduction

The ability of sensory systems to adjust its function continuously according to prior experiences represents a type of functional plasticity. Visual adaptation is one of the most attractive phenomena reflecting this adjustment. In the visual system, it is observed throughout the visual pathway, including the retina^1–5^, lateral geniculate nucleus (LGN)^6–10^, primary visual cortex^11–15^, extrastriate cortex^16–22^ and other higher-level visual areas^23–25^.

Beyond the widely existing adaptation effect in individual visual regions, it is still an open question on how the adaptation propagates across different visual regions^24, 26^. A recent electrophysiological study has found the enhanced effects of spatial adaptation from lateral geniculate nucleus (LGN) to V1^10^. Other electrophysiological studies suggest that MT inherits the direction adaptation from V1 along the primate motion/direction stream^16–18^. However, various adaptation effects were also found throughout the visual system in some fMRI studies^27–30^. For example, for contrast adaptation, the associated visual cortices along the form pathway display either similar or opposite adaptation effect from bottom to up level^27^. When adapting different features (such as contrast, orientation, direction and spatial position), the inherited adaptation effects in the downstream regions could be different.

Adaptation effects induced by certain adapting features are sometimes region-specific. The orientation adaptation induces robust decreases in firing rate and shifts in the preferred angle in area 17 recorded by both intrinsic signal imaging and single-unit recordings^13^, but the orientation-specific adaptation is weak^9, 31^ or undetectable^6, 32^ in LGN. A fMRI study also demonstrates face adaptation mostly occurs in mid-fusiform but not in the calcarine and posterior fusiform^33^. It is possible that the propagation of adaptation effect is region/feature-specific besides following the hierarchical structure of the visual system.

The appropriate sites to examine this idea in cats are area 21a and posteromedial lateral suprasylvian (PMLS), which are the gateways of form/orientation and motion/direction, respectively^34^. In fact, the propagation of adaptation effect remains unclear at the neuronal level in the form/orientation pathway. Thus we investigated the effects of orientation and direction adaptation in area 17, as well as in area 21a and PMLS. Several recording techniques including intrinsic signal optical imaging and single-unit recordings were applied to examine the hemodynamic signals, as well as action potentials, since some of the previous contradictory results were reflected in different signal sources. Based on different adapting stimuli (drifting and flashing gratings, as well as moving random dots), the adaptation effect in different visual areas of cats was evaluated with two distinct adaptation protocols. We found the adaptation effect in area 21a induced by different protocols and recording methods was greater than that in area 17 with feature specificity: the enhanced adaptation effects were orientation-specific along the form/orientation pathway, while direction adaptation was enhanced from area 17 to PMLS but not to area 21a.

## Results

### Electrophysiologically recorded adaptation effects in areas 17 and 21a induced by top-up protocol

**Figure 1:**
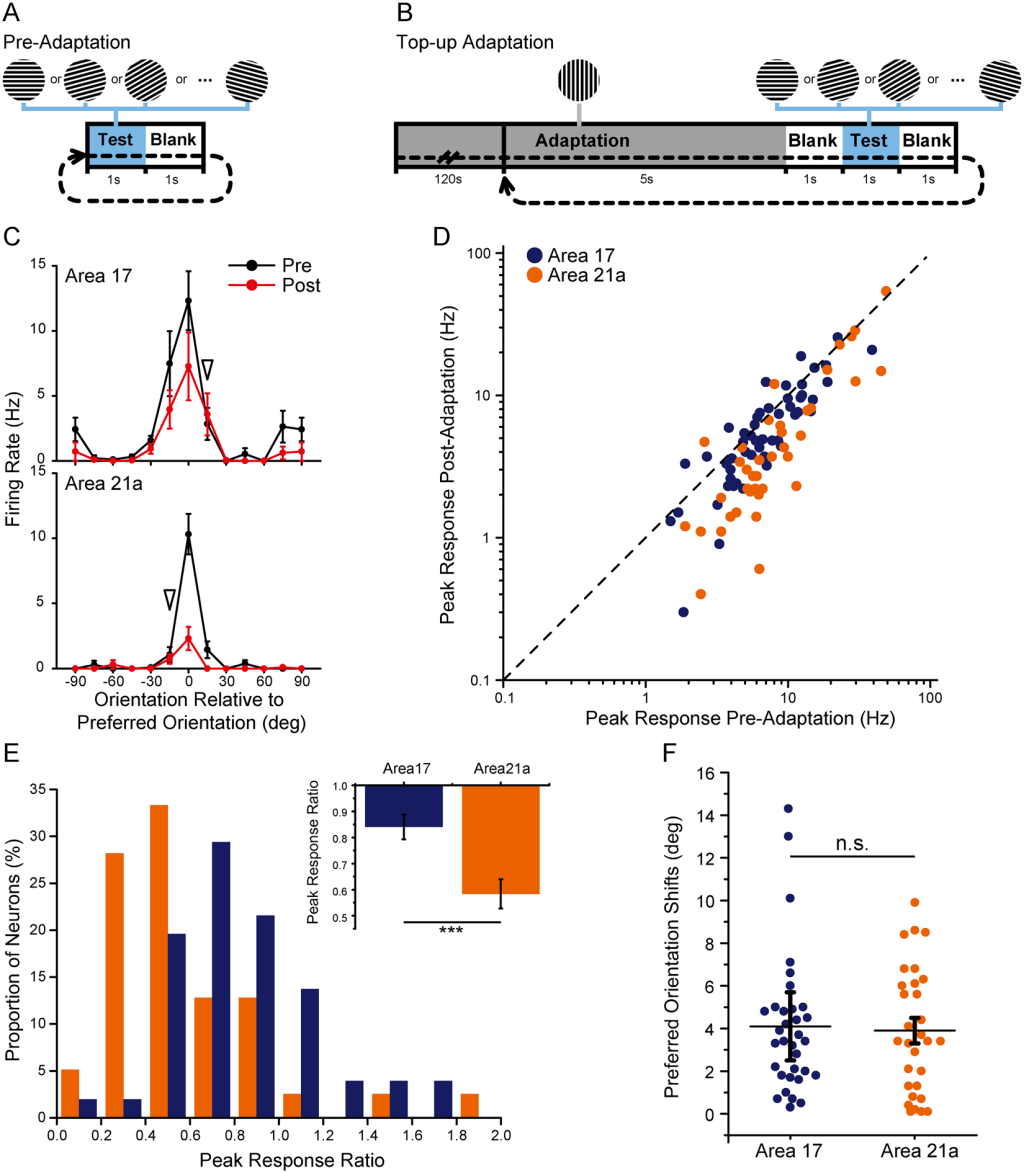
Top-up orientation adaptation in areas 17 and 21a measured by single-unit recordings. (**A,B**) Protocols before (**A**) and during top-up adaptation (**B**). (**C**) Orientation-tuning curves of example neurons in area 17 (top) and area 21a (bottom) before (black lines) and after adaptation (red lines). Arrowhead indicates the orientation of the adapter. Error bars indicate standard error of mean (SEM, n = 20 repeats). (**D**) A comparison of the peak response before and after adaptation in area 17 (blue dots) and area 21a (orange dots). (**E**) Distribution histograms of the peak response ratios (Post-Adaptation/Pre-Adaptation) in area 17 and area 21a neurons. Up corner: Statistics of the peak response ratios in area 17 (blue bar, n = 51) and area 21a (orange bar, n = 39, p = 0.0004, t-test). (**F**) Preferred orientation shifts of area 17 (blue dots, n = 33) and area 21a (orange dots, n = 31) neurons. Error bars indicate SEM. ***p < 0.001.

To induce an orientation adaptation effect, we performed a common top-up adaptation protocol as previously reported^13, 15, 17, 18, 35^ in both areas 17 and 21a. Before adaptation, the test stimuli were presented in 24 directions with an interval of 1 s (Fig. 1A), and there was no significant difference between the mean firing rates of neurons we recorded in area 17 (mean ± SEM: 8.5 ± 1.0 Hz, n = 51) and area 21a (10.6 ± 1.7 Hz, n = 39; p = 0.26, t-test), and the orientation selectivity of neurons in area 21a (OSI: 0.63 ± 0.03, n = 39) was slightly and non-significantly greater than that in area 17 (OSI: 0.57 ± 0.03, n = 51; p = 0.066, t-test). These data were consistent with previous reports^34, 36^. As adaptation stimuli (15-deg away from the preferred orientation), a 5-s top-up adaptation and a 1-s blank were added before the test stimuli in each block, following the initial 120-s adaptation (Fig. 1B). As typical neurons showed (Pre-Adaptation: black lines, Post-Adaptation: red lines, Fig. 1C), the top-up adaptation protocol induced an adaptation effect, including a prominent decline in the neuron’s firing rate and a slight preferred orientation shift in both area 17 (top) and area 21a (bottom).

To evaluate the response reduction, we compared the peak response in the preferred orientation before and after adaptation in both area 17 (blue dots) and area 21a (orange dots, Fig. 1D), and most of the neurons showed reductions in their responses after adaptation (below the diagonal line, Fig. 1D). The peak response ratio was further defined as the ratio of the neuron’s peak response after adaptation to that before adaptation (see Methods). The distributions of peak response ratios in area 17 (blue bars) and area 21a (orange bars) were significantly different (p < 0.0001, Kolmogorov-Smirnov test; Fig. 1E). On average, the peak response ratios of area 21a (0.58 ± 0.06, n = 39) were significantly smaller than those of area 17 (0.84 ± 0.05, n = 51; p = 0.0004, t-test; Fig. 1E), suggesting an enhanced adaptation effect from area 17 to area 21a.

In our data, we observed that orientation selectivity index decreased slightly after adaptation in both area 17 (OSI: 0.52 ± 0.03, n = 51; p = 0.034, paired t-test) and area 21a (OSI: 0.55 ± 0.04, n = 39; p = 0.0047, paired t-test). To evaluate the relationship between the orientation adaptation effect and a neuron’s orientation selectivity, we compared the correlations between OSI and peak response ratios, but we observed no significant correlation in either area 17 (correlation coefficient r = −0.052, p = 0.72, n = 51, Pearson test) or area 21a (correlation coefficient r = −0.012, p = 0.46, n = 39, Pearson test). We further examined neurons with a high OSI (top 30%), and found the peak response ratios for area 21a (0.59 ± 0.08, n = 12) were still significantly smaller than those for area 17 (0.86 ± 0.09, n = 14; p = 0.02, t-test). To compare the adaptation effects in different cell types, we categorized the neurons into fast-spike (FS) and regular-spike (RS), based on the ratio of the second and first peak amplitude in the recorded spike waveform (P2/P1, FS neurons: P2/P1 > 0.5; RS neurons: P2/P1 < 0.5.), as previously reported^37^. Our data showed that the peak response ratios in area 21a were significantly smaller than those in area 17 in both FS and RS neurons (FS neurons: area 17, 0.95 ± 0.08, n = 19 vs area 21a, 0.46 ± 0.07, n = 8; p = 0.0004; RS neurons: area 17, 0.78 ± 0.06, n = 32 vs area 21a, 0.61 ± 0.07, n = 31; p = 0.037, t-test). In general, the enhanced adaptation effect between areas 17 and 21a occurred regardless of orientation selectivity and FS/RS cell type.

Furthermore, the adaptation effect difference was found in the response evoked by preferred orientation as well as orientations 15-deg away (Supplementary Table 1). It seems the orientation adaptation in area 21a was enhanced, as long as the adaptation effect was induced. Similar to area 17 and other regions in the visual system^11, 38^, the 15-deg offset orientation adaptation also led to a shift of preferred orientation in area 21a. However, there was no significant difference in the preferred orientation shifts between neurons in area 17 (4.1 ± 0.6-deg, n = 33) and area 21a (3.9 ± 0.6-deg, n = 32; p = 0.86, t-test, Fig. 1F; some neurons were not included due to a poor goodness of fit; see Methods), which were identified by our single-unit recordings (but significant in the following results by intrinsic signal optical imaging). The lack of significance may be due to the limited number of neurons.

Next, we recorded visually evoked potentials simultaneously in area 17 and 21a with the same top-up adaptation protocol. As the typical recording showed, large reductions were induced at adapting orientation (Supplementary Fig. 1A, left). In contrast, adaptation barely induced changes at non-adapting orientations (Supplementary Fig. 1A, right). We measured the absolute difference between the first positive peak and negative peak as the magnitude of the visually evoked potential signal, and calculated the ratio of peak-peak response after adaptation (Post-Adaptation/Pre-Adaptation). The response ratio in area 21a (0.73 ± 0.05, n = 26) was significantly smaller than that in area 17 (0.86 ± 0.04, n = 23; p = 0.02, t-test; Supplementary Fig. 1B). This finding demonstrated that the visually evoked potential signals reduced more in area 21a than in area 17 after orientation adaptation, and it was consistent with our findings from the single-unit recording data.

### The enhanced adaptation effect revealed by intrinsic signal optical imaging

**Figure 2:**
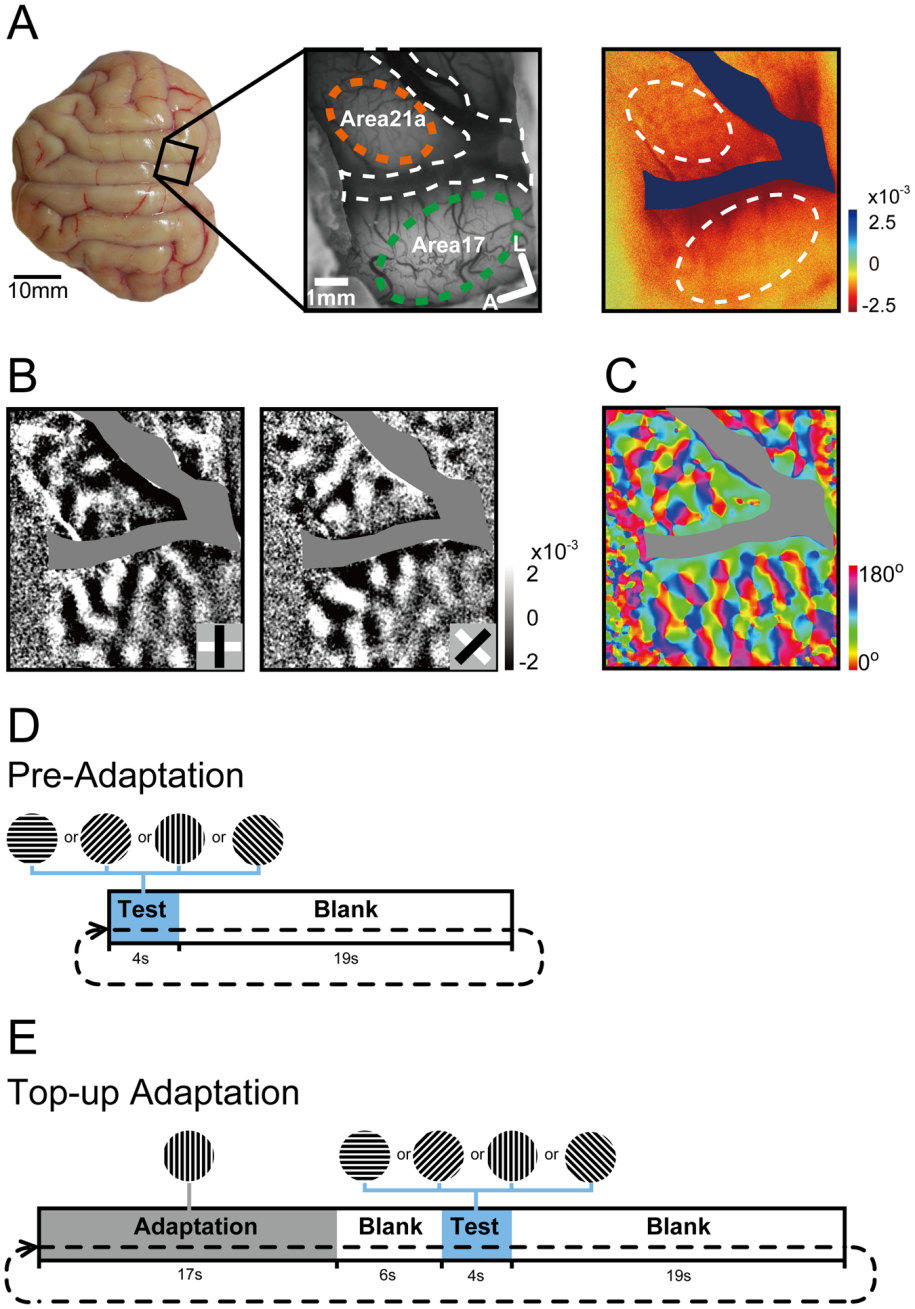
Adaptation protocol and evoked functional maps in area 17 and 21a measured by intrinsic signal optical imaging. (**A**) Craniotomy location (left, scale bar, 10 mm) of the recording window, the blood vessel map (middle, scale bar, 1 mm) and single condition map (right) of the imaged area covering both parts of area 17 (dashed line in green) and area 21a (dashed line in orange). The interfering blood vessel (dashed line in white) was identified and excluded. A: anterior; L: lateral. (**B**) The differential maps obtained by subtracting each of the two orthogonal orientation single condition maps (left: 0–90; right: 45–135). The gray scale indicates the strength of mapping signal. (**C**) The color-coded preferred orientation map based on vector summation (see Methods). The scale bar indicates the angle of preferred orientation. (**D,E**) Visual stimulus protocols before (**D**) and during top-up adaptation (**E**).

Given the spatial limitation of a single-unit recording, we sought to investigate the orientation adaptation effect with optical imaging of intrinsic signals to obtain information on a large population of neurons in areas 17 and 21a together. A large transparent window was carefully implanted above areas 17 and 21a (bold square, Fig. 2A, left) to collect and then compare the intrinsic signals (Fig. 2A, right) and orientation maps (Fig. 2B, orientation differential maps; Fig. 2C, color-coded preferred orientation map) for the two areas simultaneously.

Since the intrinsic signal optical imaging measures modulations in local oxygen consumption and blood flow, the time course of the optical imaging signals is slower than those of electrophysiological recordings, and is similar to those of fMRI^39^. We adjusted the top-up adaptation protocol based on the long-term top-up protocol in a fMRI study^40^. Before adaptation, each block of optical imaging consisted of a 4-s test stimulus and a subsequent 19-s blank (Fig. 2D). In top-up adaptation, each block of optical imaging consisted of a 17-s adapting stimulus (90-deg orientation grating drifting at 0-deg direction) plus a 6-s blank period, and a 4-s test stimulus plus a 19-s blank period (Fig. 2E). The intrinsic signals were evoked by test stimulus and collected around test period. To determine the position of the 17/18 border and confirm area 21a, we imaged cortical signals using gratings of varying spatial frequencies. An orientation differential map was obtained with the presentation of low spatial frequency gratings (0.1cyc/deg, Supplementary Fig. 1C, second column) and high spatial frequency (0.5cyc/deg, Supplementary Fig. 1C, third column). Consistent with previous reports^41^, the location of the 17/18 border (red dash line) is clearly differentiated by subtraction of the low (sum of all single orientation maps) and high spatial frequency responses (Supplementary Fig. 1C, fourth column). In this manuscript, we delineate the ROI of area 17 for calculating the global signals. Since the area 21a and area 17 are separated by sulcus lateralis (yellow bar in first column, Supplementary Fig. 1C same as Fig. 2A.) with a large blood vessel in between, they were readily distinguishable. To further clarify the area 21a functionally, we measured the distance between pairs of neighboring pinwheel centers of opposite types in 7 cats (clockwise and counterclockwise)^42^. The distribution histograms (pre-adaptation conditions) indicated that 41.9% and 62.6% of pinwheel centers had their distance longer than 500 um in area 21a (orange bars) and area17 (blue bars), respectively (on average: area 17, 559.66 ± 8.71 um, n = 430 pairs; area 21a, 480.58 ± 14.56 um, n = 124 pairs, p < 0.0001, t-test, Supplementary Fig. 1D corner). As previously reported^43^, the orientation maps were denser in 21a compared with those in area 17 (Chi-squared test, P = 0.002, Supplementary Fig. 1D). The above tendency was previously reported from electrophysiological data^44^ and optical imaging signals^43, 45^. In summary, we confirmed the imaging site of area 21 and area17 in multiple ways. To validate this protocol, we first measured its adaptation effect by single-unit recordings in area 17. We found that this long-term top-up adaptation protocol could induce significant adaptation in all the tested neurons (below the diagonal line, Supplementary Fig. 2A). The peak response ratios were smaller than 1 (on average: 0.53 ± 0.08, n = 10; p < 0.0001, t-test), while they were approximately 1 with a blank (mean ratio: 1.03 ± 0.07, n = 18, p = 0.64, t-test) or a random-orientation adaptation protocol (mean ratio: 1.05 ± 0.13, n = 12, p = 0.72, t-test). These data confirmed that the long-term top-up protocol for optical imaging was appropriate to induce an adaptation effect of a proper intensity at single cell level.

**Figure 3:**
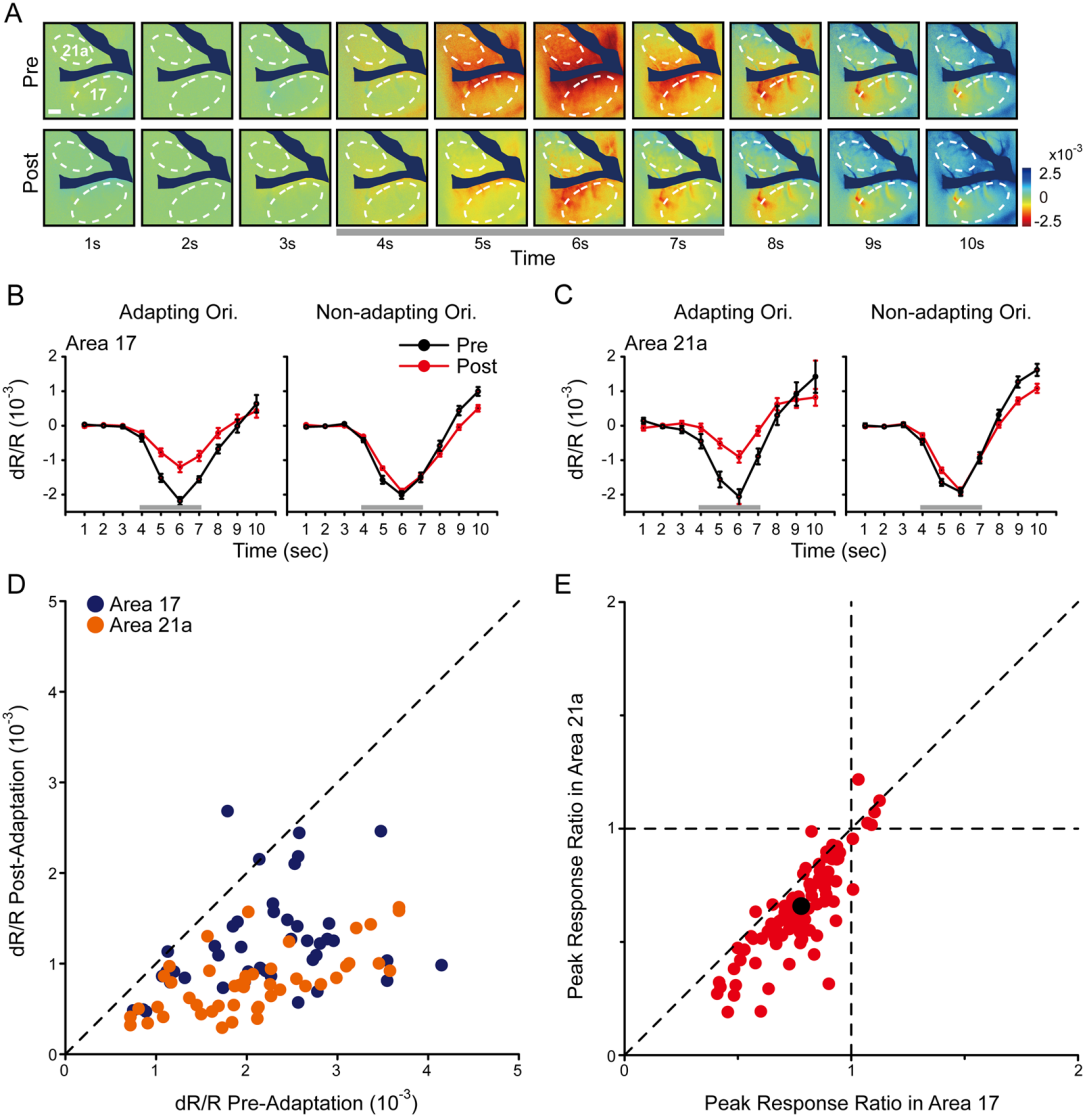
Top-up orientation adaptation in areas 17 and 21a measured by intrinsic signal optical imaging. (**A**) The single-condition maps before (top) and after adaptation (bottom). The drifting gratings were presented from the 4th to the 7th second. Bottom gray bar: the period of stimulus presentation. Scale bar, 1 mm. (**B,C**) The time-course curve of the global signals before (black lines) and after adaptation (red lines). The mean dR/R of the exposed areas 17 and 21a was calculated for each frame in (**A**), and the curves for adapting orientation (left) and non-adapting orientation (right) were compared. Standard errors are illustrated (n = 14 trials with 42 repeats). Error bars indicate SEM. (**D**) The peak global signals (from 6th frame) before and after adaptation in area 17 (blue dots) and area 21a (orange dots). (**E**) The ratios of the peak global signals (Post-Adaptation/Pre-Adaptation, see Methods) were compared between areas 17 and 21a as pairs. It is notable that areas 17 and 21a from the same animal were simultaneously imaged and compared. Each dot represents one imaging point from a chronic transparent window on a given day. Black dot indicates mean with SEM (n = 95 imaging points from 9 cats, p < 0.0001, paired t-test).

By comparing the single-condition maps elicited by the adapting orientation before (top) and during top-up adaptation (bottom, Fig. 3A), we found that the response was considerably reduced in both areas 17 and 21a. This finding was in accordance with our results obtained with single-unit recordings and visually evoked potentials. To quantify the response reductions, we analyzed the global signals in the region of interest (Fig. 3A, dotted lines), which is similar to the analysis on BOLD signals of fMRI^46, 47^, to characterize the population responsiveness of the neurons. As seen in Fig. 3B,C, the global signals of non-adapting orientations were not influenced by orientation adaptation (Fig. 3B,C, right), while those of adapting orientations were reduced in area 17 (Fig. 3B, left) and area 21a (Fig. 3C, left). We then quantified the adaptation effect with a scatter plot of the global signal peak value (from the 6th frame evoked by adapting orientation). After adaptation, the reduction in area 21a (orange dots) seemed to be greater than that in area 17 (blue dots, Fig. 3D). In terms of the peak response ratio (Fig. 3E, each dot represents simultaneously recorded data from areas 17 and 21a of the same cat in one imaging session, see Methods), most of the ratios were smaller than 1, which demonstrated that the adaptation caused a response decline in both areas. More importantly, the data points were mostly located below the diagonal line, showing a greater reduction in area 21a. On average (black dot in Fig. 3E, mean with SEM), the response ratios in area 21a (0.66 ± 0.021) were smaller than those in area 17 (0.78 ± 0.016; n = 95 imaging points from 9 cats; p < 0.0001, paired t-test; Fig. 3E; also significant across animals: area 17, 0.76 ± 0.030 vs area 21a, 0.63 ± 0.036, n = 9 cats; p < 0.0001, paired t-test). These results demonstrated that the global signals of adapting orientation were specifically reduced in both areas, and importantly, a greater response reduction occurred in area 21a than in area 17 when top-up adaptation was introduced.

Another concern on the protocol was that the response reduction was due to the prolonged 17-s stimuli and subsequent insufficient recovery of the slow hemodynamic signals. If so, no matter what the adapter’s orientation was, there would be non-specific response reduction. However, in Fig. 3B,C, the reduction was clearly orientation specific: with the prior 17-s adapting-orientation stimulus, the test stimulus drifting at non-adapting orientations evoked unchanged global signals (right panels). To further confirm it, we designed control top-up adaptation protocols, by using randomly presented 4 orientations drifting gratings (Supplementary Fig. 2B, top, random orientation adaptation) or blank screen as adapting stimuli (Supplementary Fig. 2B, bottom, blank adaptation), and found that they could not induce significant response reduction (Supplementary Fig. 2C, random orientation adaptation: gray bars; blank adaptation: forward-slash bars; normal adaptation: red bars). On average, the peak response ratios with normal adaptation (0.78 ± 0.016, n = 95 imaging points) were significantly smaller than those with blank or random orientation adaptation (normal vs blank, 1.02 ± 0.014, n = 32 imaging points, p < 0.0001; normal vs random, 0.98 ± 0.02, n = 52 imaging points, p < 0.0001, t-test), but not between the blank and random adaptation (p = 0.18, t-test; Supplementary Fig. 2C, right top). These results further demonstrated that the global signals were well and specifically adapted.

Since top-up adaptation is a temporally dynamic procedure, we wondered how this difference occurred across time. For this purpose, we performed 3 sequential top-up adaptation sessions (Supplementary Fig. 3A, each session lasted for 37 min, and a short break about 2 minutes was inserted in between). In four typical cases (Supplementary Fig. 3B), as well as in the average (Supplementary Fig. 3C, n = 9 cats), the adaptation effect was enhanced progressively across time. Importantly, the adaptation in area 21a (orange lines) was always greater than that in area 17 (blue lines) throughout the adaptation procedure.

**Figure 4:**
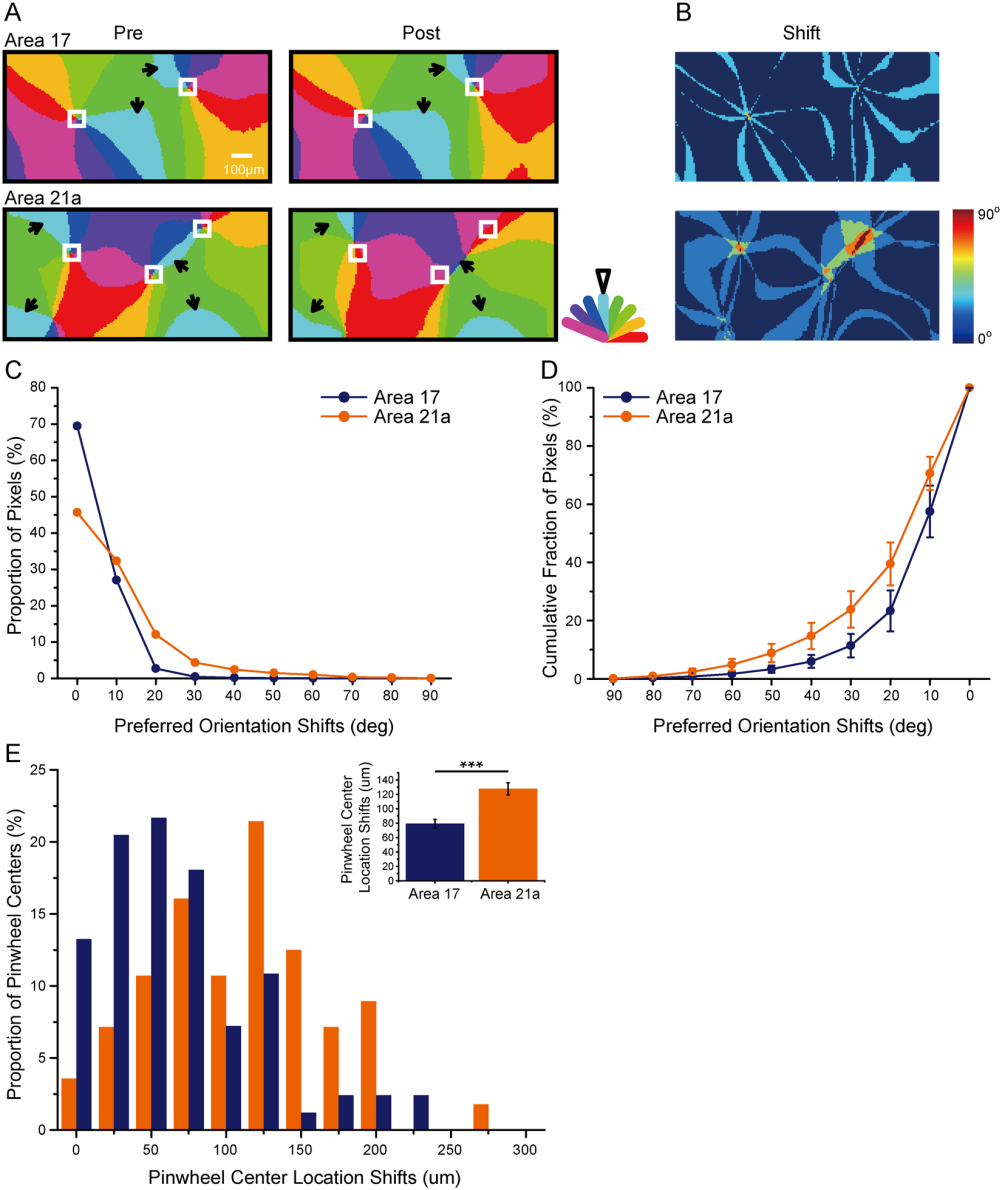
Adaptation effect on preferred orientation maps in areas 17 and 21a. (**A**) Examples showing the adaptation-induced shifts of preferred orientation maps in area 17 (top) and area 21a (bottom). In the preferred orientation maps obtained before (left) and after adaptation (right), the boundaries of the adapting orientation domains (solid arrowheads) and the pinwheel centers were located (white boxes). Adapting orientation is indicated by an open arrowhead in the color bar. Scale bar, 100 μm. (**B**) Shifts of preferred orientation maps obtained by subtracting the preferred orientation maps in (**A**). (**C**) The distribution histograms of the preferred orientation shifts in area 17 (blue) and area 21a (orange) from one cat. (**D**) The cumulative fraction of the preferred orientation shifts from 5 cats. Error bars indicate SEM. Area 17 vs area 21a: p < 0.0001, Kolmogorov-Smirnov test. (**E**) The shifts of pinwheel center locations in area 17 (blue bars) and area 21a (orange bars). For each pinwheel center in the Pre-adaptation preferred orientation map, the nearest pinwheel center in the post-adaptation map was located, and their spatial distance was calculated. Top corner: the shifts in the pinwheel center locations in area 21a were significantly larger than those in area 17 (p < 0.0001, t-test). Error bars indicate SEM.

As previous studies reported^13, 48^, orientation adaptation influences the orientation maps of the primary visual cortex in cats, but the map shift in area 21a remains unknown. To investigate the spatial properties of the orientation adaptation, we measured the preferred orientation maps in areas 17 and 21a before (Fig. 4A, left) and during top-up orientation adaptation (Fig. 4A, right). Similar to previous reports in area 17, the adapting orientation domains shrunk, and a notable change in area 17 was observed. However, the shift in area 21a seemed to be stronger (light blue regions in Fig. 4A). This tendency was more evident in the absolute shift maps (Fig. 4B) when the preferred orientation maps obtained before and after adaptation were subtracted. Then, we compared the absolute shifts in the two areas quantitatively (Fig. 4C, a typical distribution of the absolute shifts from one cat). In both areas, the majority of shifts were not greater than 30-deg, which was consistent with previous studies of single-unit recordings^13, 49^. More importantly, the shifts in area 21a seemed to be greater than those in area 17 (Fig. 4C), which was further confirmed by cumulative curve of shifts in the two areas (p < 0.0001, n = 5 cats, Kolmogorov-Smirnov test; Fig. 4D).

As another way to evaluate the changes in preferred orientation maps^50, 51^, we located the pinwheel centers (boxes in Fig. 4A) and measured their shifts (Fig. 4E). It seemed that the adaptation induced some shifts in the pinwheel centers in both areas (area 17: 79.35 ± 5.81 μm, n = 83; area 21a: 127.74 ± 8.44 μm, n = 57), meaning that the adaptation caused some kind of re-organization in the orientation maps (Fig. 4E). More notably, the shifts in the pinwheel center locations in area 21a were significantly larger than those in area 17 (p < 0.0001, t-test). Together, these data show that the orientation adaptation had a greater influence on the orientation maps in area 21a compared with those in area 17.

### Adaptation effect from area 17 to 21a induced by biased adaptation protocol

Adaptation effect also depend on the protocol. In addition to the top-up protocol, the adaptation effect can be elicited when the probability of spatial position or orientation stimuli is biased in the primary visual cortex^10, 52^. We wondered whether an orientation-biased stimulus would induce an orientation adaptation in area 21a and whether the difference of the adaptation effects between the two areas would still be there.

**Figure 5:**
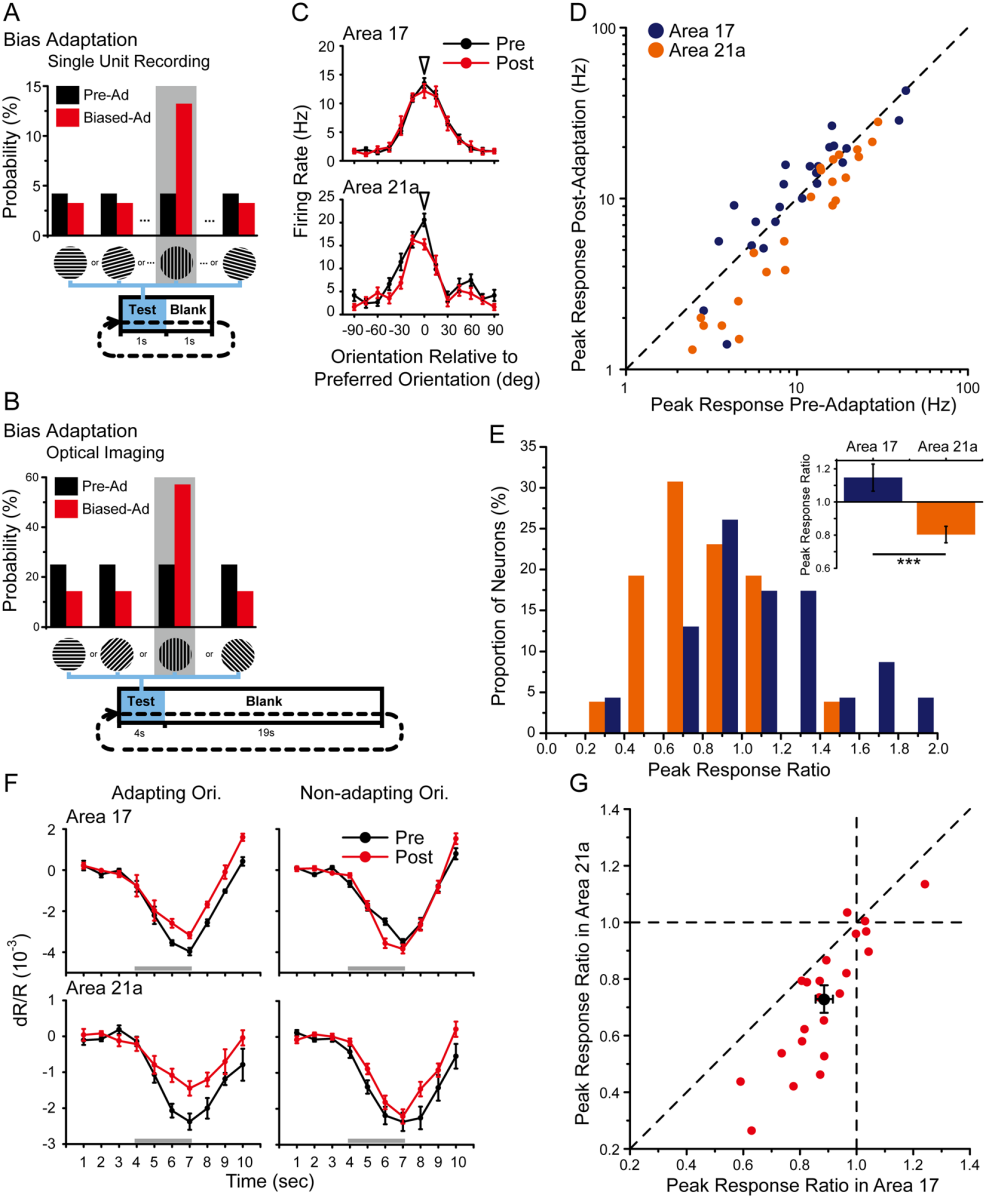
Biased orientation adaptation in areas 17 and 21a measured by single-unit recordings and intrinsic signal optical imaging. (**A,B**) Protocols before and during biased adaptation. Before adaptation, stimuli were represented with equal probability (black bars). During adaptation, the adapting orientation was presented with a probability that was 3 times more than those of the others (red bars). (**C**) Orientation-tuning curves of example neurons in area 17 (top) and area 21a (bottom) before (black lines) and during adaptation (red lines). Arrowhead indicates the orientation of the adapter. Error bars indicate SEM (n = 20 repeats). (**D**) Peak response before and after adaptation in area 17 (blue dots) and area 21a (orange dots). (**E**) Distribution histograms of the neuron peak response ratios in area 17 (blue bars) and area 21a (orange bars). Top corner: statistics of the peak response ratios in area 17 (blue bar, n = 23) and area 21a (orange bar, n = 26, p = 0.0003, t-test). Error bars indicate SEM. (**F**) The time-course curve of the global signals before (black lines) and during adaptation (red lines) in area 17 (top) and area 21a (bottom). Error bars indicate SEM from 8 trials (24 repeats). Error bars indicate SEM. (**G**) A paired comparison of peak response ratios in areas 17 and 21a from 22 imaging points in 4 cats (p < 0.0001, paired t-test). Black dot indicates mean with SEM.

We applied biased adaptation protocols in electrophysiological recordings (Fig. 5A) and optical imaging sessions (Fig. 5B; see Methods). Before the biased adaptation (black), drifting gratings at 12 (for single-unit recordings) or 4 (for optical imaging) orientations were presented randomly, and the probability of each orientation was equal. During the biased adaptation (red), one of the orientations was biased and the probability of its presence was 3 times higher than the others.

In our single-unit recordings, the response reductions caused by biased adaptation in both areas seemed to be much smaller than those caused by top-up adaptation (representative neurons in Fig. 5C and total in Fig. 5D). We plotted the peak responses before and during biased adaptation (Fig. 5D), and compared the distributions and means of the peak response ratios in the two areas (Fig. 5E). A significant difference was found between their distributions (p = 0.01, Kolmogorov-Smirnov test; Fig. 5E), and the peak response ratios in area 21a (0.80 ± 0.05, n = 26, orange bars) were still significantly smaller than those in area 17 (1.15 ± 0.08, n = 23, blue bars) in our comparison (p = 0.0003, t-test). In fact, in area 17, there was no significant adaptation, while the same protocol induced significant orientation adaptation in area 21a.

We also investigated the biased adaptation effect using optical imaging to collect neuronal responses from a larger population in the two areas (representative global signals in Fig. 5F). The same measurements and analyses were applied as those used for top-up adaptation. For vast majority of paired areas recorded simultaneously, the peak response ratios in area 21a were smaller than those in area 17 (Fig. 5G, below the diagonal line); the average response ratio (black dot: mean with SEM) was 0.73 ± 0.049 in area 21a and 0.89 ± 0.031 in area 17 (n = 22 imaging points from 4 cats; p < 0.0001, paired t-test; Fig. 5G; comparison across animals: area 17, 0.88 ± 0.015 vs area 21a, 0.72 ± 0.030, n = 4 cats; p = 0.01, paired t-test).

We noticed that the adaptation effects in area 17 identified by single-unit recordings (not significant) and optical imaging (significant) were different in this protocol. This might have been due to a larger number of non-adapting orientations in the overall single-unit recordings (only 1 out of 12 orientations was an adapter, while 1 of 4 orientations was an adapter in optical imaging). Thus, we increased the probability of the adapter by 20 times in a single-unit recording from another set of experiments. The results showed that this strongly biased protocol induced significant adaptation in area 17 (0.88 ± 0.04, n = 9). Importantly, the adaptation effect induced by the same strong protocol was also greater in area 21a (0.73 ± 0.04, n = 15) than that observed in area 17 (p = 0.013, t-test).

In summary, similar to the effect induced by the top-up protocol, the orientation adaptation observed in area 21a was induced by a biased adaptation protocol, and the orientation adaptation effect in area 21a was still greater than that in area 17.

### The feature-specific propagation of adaptation

The above results demonstrated an enhanced adaptation effect in area 21a, which is the gate of the ventral pathway decoding form/orientation information in the cat visual system^53^. Next, we examined how motion/direction information, which is mostly processed along dorsal visual pathway, was adapted in areas 17 and 21a, and thus to examine the feature-specificity of the adaptation propagation.

Since drifting gratings we used above contained both orientation and direction information, and they may have contaminated each other, we applied flashing gratings and moving random dots as the test and adapting stimuli to observe orientation-specificity and direction-specificity of the enhanced adaptation.

**Figure 6:**
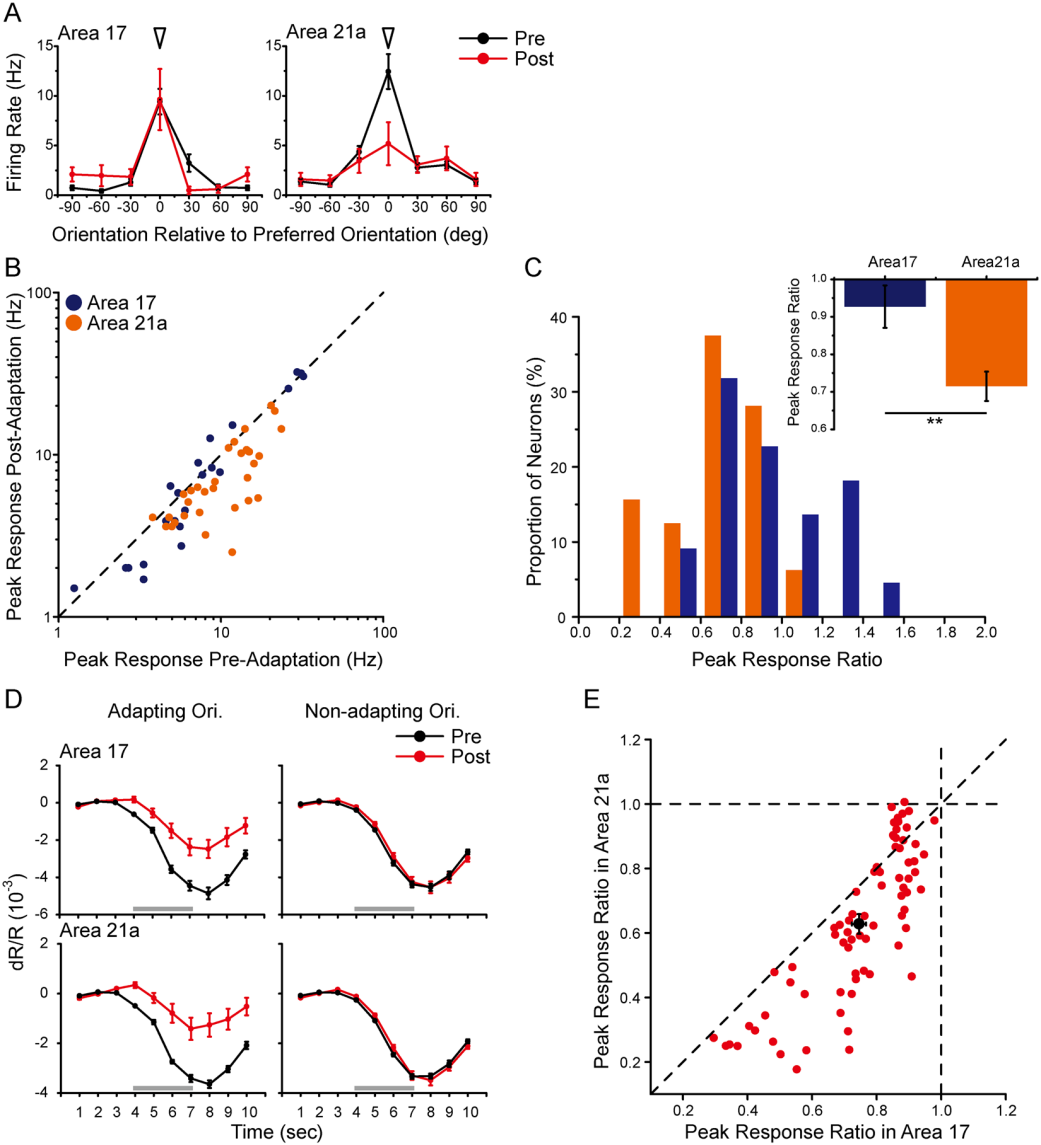
Orientation adaptation induced by stationary flashing gratings and measured by single-unit recordings (**A–C**) and intrinsic signal optical imaging (**D,E**) in areas 17 and 21a. (**A**) Orientation-tuning curves of example neurons in area 17 (left) and area 21a (right) before (black lines) and after adaptation (red lines). Arrowhead indicates the orientation of the adapter. Error bars indicate standard error of mean (SEM, n = 20 repeats). (**B**) A comparison of the peak response before and after adaptation in area 17 (blue dots) and area 21a (orange dots) measured by single unit recordings. (**C**) Distribution histograms of the peak response ratios in area 17 and area 21a neurons. Top corner: statistics of the peak response ratios in area 17 (blue bars, n = 22) and area 21a (orange bars, n = 32, for comparison, p = 0.001, t-test). Error bars indicate SEM. (**D**) The time-course curve of the global signals before (black lines) and after adaptation (red lines). The curves for adapting orientation (left) and non-adapting orientation (right) were compared. Standard errors are illustrated (n = 8 trials with 24 repeats). (**E**) A comparison of the peak response ratios in areas 17 and 21a measured by optical imaging (n = 74 imaging points, p < 0.0001, paired t-test). Black dot indicates mean with SEM.

Stationary flashing gratings were presented in the top-up orientation adaptation protocol. By single-unit recordings, a typical neuron showed (Pre-Adaptation: black lines, Post-Adaptation: red lines, Fig. 6A) a slight decline in the neuron’s firing rates in area 17 (left), and a prominent reduction in area 21a (right). In both area 17 (Fig. 6B, blue dots) and area 21a (orange dots), most of the neurons showed reductions in their peak responses after adaptation (below the diagonal line). The distributions of peak response ratios in area 17 (blue bars) and area 21a (orange bars) were statistically different (p = 0.04, Kolmogorov-Smirnov test; Fig. 6C). On average, the peak response ratios of area 21a (0.71 ± 0.04, n = 32) were significantly smaller than those of area 17 (0.93 ± 0.06, n = 22; p = 0.001, t-test; Fig. 6C). For optical imaging, an adaptation-induced reduction occurred in the adapting orientation (Fig. 6D, left) but not in the non-adapting orientations (Fig. 6D, right) in both areas 17 and 21a. In terms of the peak response ratio in the adapting orientation (Fig. 6E), there was significant difference between the two areas (area 17, 0.75 ± 0.02 vs area 21a, 0.63 ± 0.03, n = 74 imaging points, p < 0.0001, paired t-test).

**Figure 7:**
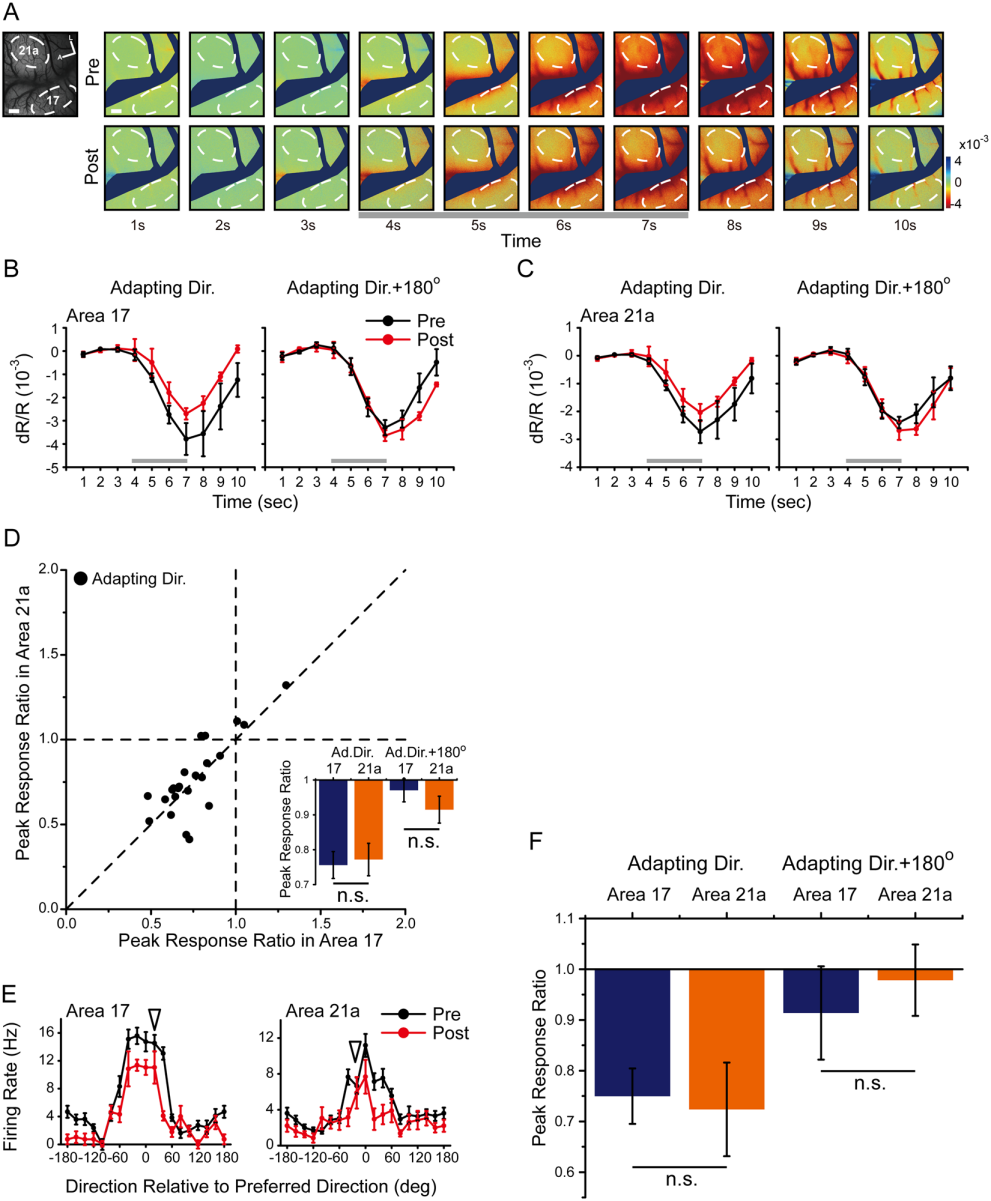
Direction adaptation induced by moving random dots and measured by intrinsic signal optical imaging in areas 17 and 21a. (**A**) Single-condition maps induced by the adapting direction before (top) and after direction adaptation (bottom). The temporal sequence of the moving random dot adaptation protocol was identical to the top-up protocol with drifting gratings. Scale bar, 1 mm. (**B,C**) The time-course curve of the global signals before (black lines) and after direction adaptation (red lines) at the adapting direction (left) and opposite direction (right). Error bars indicate SEM (n = 14 trial with 42 repeats). (**D**) A paired comparison of peak response ratios in areas 17 and 21a at adapting direction. Bottom corner: the statistics of area 17 (blue bars) and area 21a (orange bars). Left to right: peak response ratio in the adapting direction (n = 23 imaging points in 4 cats, p = 0.58, paired t-test), in the opposite direction (p = 0.19, paired t-test). Error bars indicate SEM. (**E**) Direction-tuning curves of example neurons in area 17 (left) and area 21a (right) before (black lines) and after moving random dots direction adaptation (red lines). Arrowhead indicates the direction of the adapter. Error bars indicate SEM (n = 10 repeats). (**F**) The statistics of area 17 (n = 11, blue bars) and area 21a (n = 15, orange bars). Left to right: peak response ratio in the adapting direction (p = 0.82, t-test), opposite direction (p = 0.58, t-test). Error bars indicate SEM.

We next applied the moving random dots stimuli in the top-up direction adaptation protocol (see Methods). The single-condition maps (Fig. 7A, elicited by the adapting direction) and global signals (Fig. 7B,C) were obtained before and during top-up direction adaptation. An adaptation-induced reduction occurred in the adapting direction (Fig. 7B,C, left) but not in the opposite direction (Fig. 7B,C, right) in both areas 17 and 21a, suggesting a direction-specific adaptation effect. However, in terms of the peak response ratio in the adapting direction, there was no difference between areas 17 and 21a (area 17, 0.76 ± 0.039 vs area 21a, 0.77 ± 0.047, n = 23 imaging points in 4 cats; p = 0.58, paired t-test; Fig. 7D left; across animals: area 17, 0.76 ± 0.033 vs area 21a, 0.79 ± 0.063, n = 4 cats, p = 0.45, paired t-test).

In addition, a few single-unit recording data were obtained for moving random dots protocol (two typical neurons in Fig. 7E). There was a significant response reduction in the adapting direction. However, the mean peak response ratios (Fig. 7F) were similar in areas 17 and 21a (adapting direction: area 17: 0.75 ± 0.05, n = 11; area 21a: 0.72 ± 0.09, n = 15, p = 0.82; opposite direction: area 17: 0.91 ± 0.09; area 21a: 0.98 ± 0.07, p = 0.58, t-test).

In summary, when adapting the orientation information with stationary flashing gratings, the adaptation in area 21a was significantly greater than that in area 17, while adaptation effects were similar when adapting the direction information with moving random dots. These results demonstrated that the propagation of adaptation effect from area 17 to 21a was feature-specific and may relate closely to the visual information that the visual stream carried.

In contrast to the form stream, the motion/direction pathway is considered to process motion-related information. Since the enhanced adaptation effect along the form pathway (from areas 17 to 21a) was orientation-specific but not direction-specific, we speculated that the adaptation effect along the motion pathway would be greater when adapting direction information. PMLS was regarded as the entrance of the motion pathway in cats^53^, and therefore, we sought to investigate the direction adaptation effects in PMLS and compare the effects between area 17 and PMLS.

Our preliminary visually evoked potential and single-unit recording results of top-up direction adaptation in area 17 and PMLS (difficult to image by optical imaging due to a large unexposed cortical surface) using moving random dots are shown in Supplementary Fig. 4. In PMLS, a direction-specific reduction in responsiveness was observed in the typical cases by visually evoked potentials (Supplementary Fig. 4A) and single-unit recordings (Supplementary Fig. 4B). On average, the direction adaptation-induced reduction in PMLS (peak response ratio of visually evoked potential: 0.85 ± 0.08, n = 12, gray bar in Supplementary Fig. 4C; peak response ratio of single-unit recordings: 0.52 ± 0.06, n = 17, gray bar in Supplementary Fig. 4D) was significantly greater than that in area 17 (visually evoked potential: 1.03 ± 0.04, n = 21, p = 0.016; single-unit recordings: 0.75 ± 0.05, n = 11, p = 0.005, t-test, blue bar) and area 21a (visually evoked potential: 1.08 ± 0.06, n = 28, p = 0.02; single-unit recordings: 0.72 ± 0.09, n = 15, p = 0.03, t-test, orange bar), while there was still no difference between those in areas 17 and 21a (visually evoked potential: p = 0.51; single-unit recordings: p = 0.82, t-test). In the preliminary results, we observed an enhanced direction adaptation along the motion stream from area 17 to PMLS, but not along the form stream. Although more convincing data along dorsal/motion pathway are still needed in future studies, these electrophysiological results suggested a feature-specificity of the adaptation propagation along the two visual streams.

## Discussion

The adaptation effect in the visual system is complicated and sometimes controversial^24^, partially due to various recording techniques and adaptation protocols. Trying to minimize their possible influences, we investigated orientation adaptation in area 21a together with area 17 using two distinct adaptation protocols (top-up and biased), three different stimuli (drifting gratings, flashing gratings and moving random dots), and multiple techniques (single-unit recording, visually evoked potential and intrinsic signal optical imaging), to examine the propagation of adaptation from area 17 to area 21a. Different protocols may reflect different aspects of adaptation. For example, the top-up adaptation protocol focuses on the aftereffect induced by a prior adaptation^24^ and is particularly appropriate for the analysis of response dynamics after adaptation. As for biased adaptation, the probabilities of stimuli are crucial, and the adaptation is a statistical result of the massive stimuli that can explain the population homeostatic maintenance of adaptation^52^. Thus, these protocols have been applied at different visual regions, including the LGN^8, 10^, primary visual cortex^15, 38, 52^ and MT^17, 18, 54^, as well as in fMRI studies^27, 30, 40, 55^.

Based on the results with above protocols, we observed diversified (temporal, spatial, response amplitude, preferred orientation shifts) adaptation effects and that the orientation adaptation was unanimously enhanced as long as the orientation adaptation was evoked in both areas 17 and 21a, suggesting that the propagation of this adaptation along the ventral stream was not protocol-dependent and might come from a general cortical mechanism.

A similar cascaded effect was also described in recent electrophysiological studies on receptive field properties between LGN and V1 in the mouse^10^ and on inherited change of direction selectivity between V1 and MT in the monkey^16^. As for orientation adaptation, there are also some hints from the literature. In the LGN, the orientation-specific adaptation effects are weak^9, 31^ or undetectable^6, 32^, while the studies in the primary visual cortex^11, 13, 14, 38, 52, 56–60^ and extrastriate cortices^18, 20, 22, 61^ found clear and robust orientation adaptation. Together, the above studies suggest an enhanced orientation adaptation starting from the LGN, and our experiment clearly demonstrated it along the ventral visual pathway in cortices.

In our optical imaging and single-unit recordings, we resolved orientation or direction specific adaptation effects with stationary flashing gratings or moving random dots stimuli, and the results showed that the orientation but not the direction adaptation effect was enhanced along the ventral/form stream from area 17 to area 21a. However, we observed an enhanced direction adaptation effect in PMLS which was considered as the gate of dorsal/motion pathway in cats. These results indicated a feature-specificity of the adaptation propagation across different brain regions. For the information of “what”, the adaptation becomes stronger along the ventral stream, and for the information of “where”, the enhancement occurs along the dorsal stream. In fact, both feature and region can play important roles in adaptation. In the individual cortex, some studies support the idea of feature-specific adaptation. For example, the adaptation effects induced by identical-face-repeating in the human fusiform gyrus (hFG) is more sensitive to the viewpoint of faces rather than the image size^25, 62^. Between different areas, region-specific adaptation was also identified. Soon *et al*. (2003) found that repeating identical faces led to a more robust adaptation effect in bilateral mid-fusiform and right prefrontal regions, but not the calcarine and posterior fusiform regions. Our study has tried to explain the feature- and region-specificity of adaptation under the well-known picture of dorsal and ventral information processing pathway.

As previously reported, a feedforward mechanism was applied to describe the enhanced adaptation effect^10^. For example, an MT neuron receives inputs from a bank of V1 neurons, and the adaptation effect in V1 will accumulate and propagate to MT^16^. The same logic may also hold true between areas 17 and 21a. Importantly, the feature-specificity in our results further suggests that special attention should also be paid to the feature-specific integration. In detail, the way in which a feature-selectivity is enhanced via selective integration of multiple inputs will also determine how feature-specific adaptation propagates.

Notably, feedback modulation exists widely in the visual system and is important for the visual system to work more precisely^63–66^. More importantly, the feedback modulation from areas at higher levels may have feature-specificity^51, 67, 68^. For instance, the modulation from PMLS mostly affects the direction map but not the orientation map in area 17^67^, while the feedback from area 21a only modulates the orientation map in area 17^51^. This feature-specific feedback may play an important role in the propagation of adaptation along the visual streams, and future studies are needed to examine this idea.

## Methods

### Study Approval

Twenty-five normal cats of either sex were used in these experiments. All experiments involving animals were carried out in accordance with the approved guidelines and all animal experimental protocols were approved by the Animal Care and Use Committee of Fudan University.

### Animal Preparation

Anesthesia was induced with ketamine hydrochloride (20 mg/kg) and sustained by 2.0–3.0% isoflurane (RWD Life Science Co., China) during the surgery. All pressure points and incised tissues were infiltrated with lidocaine. After the surgery, anesthesia was maintained with 1.0–2.0% isoflurane. Cats were paralyzed (i.v. gallamine triethiodide, 8–10 mg/kg/h) and artificially respired by a pulmonary pump (6025, UGO Basile, Italy) to maintain end-tidal CO_2_ at 3.5–4.0%. The body temperature of animals was monitored and maintained at 38.0 °C throughout the procedure by an automatic temperature control system (BME-461A, Institute of Biomedical Engineering, CAMS). Electroencephalogram (EEG) and electrocardiogram (ECG) were monitored continuously to ensure adequate anesthesia. The pupils were dilated with atropine (1%), and the nictitating membranes were retracted with neosynephrine (5%). The eyes were refracted and corrected with contact lenses.

As described in our previous reports^43, 67, 69^, a craniotomy and durotomy were performed at Horsley-Clarke coordinates P2-P10, L0-L5 for area 17 and A5-P7, L9-L17 for area 21a and PMLS to allow electrophysiological recordings^34, 70, 71^. Area 21a is located in the middle part of caudal suprasylvian gyrus defined by anatomical connections and retinotopic organization in cats^72^ and bounded medially and caudally by area 19 while laterally bordered by posteromedial lateral suprasylvian area (PMLS). These relationships were also described by van der Gucht *et al*. (2001), who identified cytoarchitectonic distinctions among visual cortices (e.g., area 17 and area 21a, PMLS, etc.)^73^. A plastic chamber was secured to the skull using dental cement. Extracellular electric signals were recorded by glass-coated tungsten microelectrodes (3–5 MΩ), and visually evoked potentials were recorded by Epoxylite-insulated microelectrodes (<1 MΩ, Metal Microelectrodes, FHC, USA). For chronic intrinsic signal optical imaging, the visual cortical area 21a and area 17 were exposed at Horsley-Clarke coordinates L7–12, P1–7 and L0–8, P0–10 respectively. In this work, a large craniotomy and durotomy were performed at Horsley-Clarke coordinates A5-P10, L0-L15 to expose both areas 17 and 21a. The large field is useful for getting a large map of high quality of areas 21a and 17 in one imaging field, and also importantly identifying the location of area 21a based on the relationship to the lateral suprasylvian and lateral sulci^72^ and precise positions of areas 17 and 18 based on functional organization of preferred orientation with different spatial frequencies gratings (Supplementary Fig. 1C–E). At the end of the experiment, an electrode was left in the targeted area 21a, and the animal was perfused and Nissel stained to reconstruct the electrode track to confirm the imaging location in area 21a. After the removal of the dura, an artificial dura (0.005-inch Silicon Sheeting, Specialty Manufacturing Inc., USA) was positioned on the surface of the exposed cortex. It was then covered with 3% agar (Type 1 Low EEO, Sigma-Aldrich, USA) in 0.9% saline and sealed with a transparent cover glass. This chronic transparent window was cemented to the skull by super dental bond (Super-Bond C&B, Sun Medical Co., Japan). After 3 days’ recovery, the cat with chronic transparent window was imaged multiple times during the following week as previously described^74^. In each imaging session, the cat was first anesthetized and then imaged to obtain a full set of data (before and after adaptation), and multiple data points (imaging points in Figs 3D,E, 5G, 6E and 7D) from one cat on different days were collected and analyzed.

### Stimuli

The visual stimuli were computer-generated using MATLAB (MathWorks, Massachusetts, USA) and a VSG graphic board (VSG 5, Cambridge Research Systems, Rochester, UK) or Psychtoolbox^75, 76^ and presented on a CRT monitor (FlexScan F931, Eizo Nanao Corporation, Japan) refreshing at 60 Hz and positioned 57 cm from the cat’s eyes. The cats were stimulated binocularly with full screen uniform drifting sinusoidal gratings (spatial frequency: 0.1–0.6 cycle/deg; temporal frequency: 1.5–2.5 Hz; contrast: 100%), or moving random dots (0.5–1.0 deg in diameter at a speed of 10–20 deg/s; dot density: 0.1–0.5 dots/deg^2^; contrast: 100%), or flashing gratings (spatial frequency: 0.1–0.6 cycle/deg; temporal frequency: 2 Hz; contrast: 100%). The parameters were adjusted according to each neuron’s averaged firing rate to optimize its response to the stimulus.

For electrophysiological recordings, the top-up adaptation protocol was similar to those used in previous reports^13, 15, 17, 18, 35^. We recorded responses to drifting gratings randomly presented in 24 directions (15-deg increments) before and during top-up adaptation under the following conditions: (1) before adaptation (Pre-Adaptation), gratings were randomly presented for 10 repetitions each for a total of 240 blocks, with 1-s test stimulus (randomly presented one of 24 drifting gratings) and 1-s blank (gray screen) for each block (Fig. 1A); (2) top-up adaptation (Post-Adaptation), following a 120-s initial adaptation, 24 directions were randomly presented as test stimuli for 240 blocks, 10 repetitions for each grating. In each block, a 5-s top-up adapting stimulus (adapter, a fixed direction drifting grating), 1-s blank, 1-s test stimulus (with random directions) and 1-s blank were presented in sequence (Fig. 1B). For the biased adaptation protocol, 24 directions were presented 1 second with equal probability before adaptation (10 repetitions for each grating) and 1-second interval blank. During the biased adaptation, 24 directions were still presented 1 second and followed by 1-second blank and the change was in the probability of each direction’s presentation: the adapting grating (one of the 24 directions) was 3 (or 19) times more likely to be presented than any non-adapting grating, 40 (or 200) repetitions for adapting grating and 10 repetitions for each non-adapting grating (Fig. 5A). In the top-up adaptation protocol with moving random dots or flashing gratings, white dots were randomly positioned and moved in 24 directions (15-deg increments) on a black background, or stationary gratings were flashed at 12 random orientations (15-deg increments) on a black background. The time course of these stimuli were in accordance with that used in drifting grating stimuli.

The details of intrinsic signal optical imaging were described in our previous reports^50, 51, 67, 43, 69^. Adjusted adaptation protocols based on those used in electrophysiological recordings were applied to induce adaptation effect in optical imaging (Supplementary Methods).

### Recordings

Single-unit signals were amplified (Dagan 2400A, Minnesota, USA), band-pass filtered (300–3 k Hz for single-unit signals) and digitized at 10 kHz using a data acquisition system (CED Micro1401, Cambridge Electronic Design Ltd., UK) under the control of spike2 software (version 6; Cambridge Electronic Design, Cambridge, England). Recordings consisted of both single-unit and multi-unit activity and were sorted offline. Spikes were sorted by spike2 software and analyzed in MATLAB.

A digital CCD camera system (1600 × 1200 pixels, 7.4 × 7.4 μm/pixel, 14 bit; PCO 1600, PCO AG, Germany) was used to record the intrinsic signals from the exposed areas 17 and 21a simultaneously. Two coupled 50-mm lenses (Nikkon 50 mm f/1.2, Nikon, Japan) were used to achieve a shallow depth of focus plane (<100 μm) to minimize blood vessel artifacts in the functional maps. Intrinsic signals evoked by the test stimuli were acquired under red light illumination (630 nm), and the focal plane was 500 μm below the pial. The CCD camera system was triggered 3 s before the test stimuli and recorded continuously for 10 s at a sampling rate of 1 Hz.

### Data Analysis

For single-unit signals, we applied a vector summation method to measure the preferred orientation and orientation selectivity index (OSI) based on the spike trains associated with each direction and orientation^43, 50, 51, 67, 69^:

1 $$S=\frac{{\sum }_{k}{r}_{k}{e}^{i2{\theta }_{k}}}{{\sum }_{k}{r}_{k}},$$

where *θ*_*k*_ is the direction of drifting grating and *r*_*k*_ is the firing rate at that orientation. The firing rate of a neuron was averaged from 50 ms to 1000 ms after the stimulus onset, and spontaneous responses were subtracted from the raw data. The preferred orientation and OSI were the phase and the amplitude of *S*, respectively.

Then, the adapting orientation was determined based on a neuron’s preferred orientation. Unless otherwise indicated, we used a ±15-deg offset from the preferred orientation as the adapting orientation.

The preferred direction and direction selectivity index (DSI) were determined using a vector summation that was similar to that used for the preferred orientation and OSI, without doubling the angle.

To precisely measure a shift in the preferred orientation, we fitted the averaged responses for each orientation before and after adaptation:

2 $${R}_{p}=a\times {e}^{-{(\frac{\theta -{\theta }_{pref}}{c})}^{2}}+b,$$

where *R*_*p*_ is the predicted response, *a* and *b* define the amplitude and baseline of the response, *c* determines the tuning width, and *θ* and *θ*_*pref*_ are the orientation variable and the preferred orientation, respectively. The goodness of fit was determined by R^2^, and cases in which R^2^ < 0.9 were excluded.

To evaluate the magnitude of response reduction induced by adaptation, the peak response ratio^11^ was defined as follows:

3 $${Peak}\,{Response}\,\mathrm{Ratio}{=}\frac{{Peak}\,{Respons}{{e}}_{{pre}}}{{Peak}\,{Respons}{{e}}_{{pre}}},$$

where *Peak Response*_*pre*_ and *Peak Response*_*post*_ are the firing rate in the preferred orientation (determined by vector summation before adaptation).

For intrinsic signals, the “first frame analysis” was applied to all raw data to remove slow noise^69, 77^, and a subsequent analysis on differential maps and global signals was performed^69^:

4 $${S}_{m}^{\ast }=\frac{{S}_{m}}{({{\sum }_{j}S}_{j})/3}-1,$$

where *S*_*m*_ and ${S}_{m}^{\ast }$ (*m* = 1, 2, 3…10) are the value of the raw data, and processed frames called single-condition maps, *S*_*j*_ (*j* = 1, 2, 3), are the first 3 frames in which the stimulus had not yet been presented (Fig. 3A).

Orientation differential maps were obtained by subtracting the two single-condition maps (Fig. 2A, right) elicited by orthogonal gratings (for example, 0 vs 90 deg, 45 vs 135 deg) to enhance the orientation-selective mapping signals. Since the strongest intrinsic signal appeared 3–4 s after the onset of the visual stimuli, we applied the subtraction on the 6th frame of the single-condition maps to generate the 90–0 deg (or 135–45 deg) differential map (Fig. 2B).

To calculate the strength of global signals, a large region of interest (ROI) covering the exposed cortex (area 17 or area 21a, >6 mm^2^ each) was selected, and the values of ${S}_{m}^{\ast }$ in the ROI for each condition (8 different directions) were averaged as the *dR/R* (Fig. 3B).

In the experiments, we obtained 4 single-condition maps evoked by gratings of different orientations uniformly distributed from 0-deg to 180-deg, and all of the 4 single-condition maps were averaged to obtain a cocktail blank. This cocktail was then subtracted from each of the 4 single-condition maps and filtered (standard deviation of 1 mm for high-pass Gaussian and 0.08 mm for low-pass Gaussian). Vector summation similar to that used for the single-unit data was performed pixel by pixel to obtain the preferred orientation maps, which were used to analyze the changes in orientation map patterns and orientation pinwheel center locations before and after adaptation (Fig. 2C).

Paired and unpaired Student’s t-test were performed to determine the statistical significance, unless otherwise indicated. P < 0.05 was considered significant at 0.05 level (*p < 0.05, **p < 0.01, ***p < 0.001).

## Electronic supplementary material

Supplementary Information
