# Supplementary material for: The feature-specific propagation of orientation and direction adaptation from areas 17 to 21a in cats
Source: Sci Rep. 2017 Mar 24;7:390. doi: 10.1038/s41598-017-00419-x (PMC5428465; doi:10.1038/s41598-017-00419-x)
Supplement: Supplementary file 1 — Supplementary Information [file 41598_2017_419_MOESM1_ESM.pdf]

Supplementary Information

Title:

The feature-specific propagation of orientation and direction adaptation from areas 17 to 21a in cats

Zhong Li*, Jianjun Meng*, Hongjian Li, Anqi Jin, Qijun Tang, Jianbin Zhu, Hongbo Yu

Methods

Adaptation protocols for intrinsic signal optical imaging.

We adjusted the top-up adaptation protocol based on the long-term top-up protocol. In brief, gratings randomly drifted in 8 directions (45-deg increments), and each grating was presented 3 times for a total of 24 blocks in a trial, with 5–20 trials presented to each subject either before or during top-up adaptation. In detail, the stimulus protocols were similar to those used in the electrophysiological recordings. However, there was a significant time lag for the intrinsic signals, which came from the hemodynamics similar to those of fMRI. Based on the long term top-up adaptation protocol in fMRI [1], we redesigned the stimulus sequence as the following: (1) before adaptation (Pre-Adaptation), each block (23-s in total) consisted of a 4-s test stimulus (randomly presented one of 8 directions drifting gratings) and a subsequent 19-s blank for recovery (Fig. 2D); (2) top-up adaptation (Post-Adaptation), a 17-s top-up adapting stimulus (adapter, 0-deg direction grating), 6-s blank, 4-s test stimulus (randomly presented one of 8 drifting gratings) and 19-s blank were presented sequentially in one block (46-s in total; Fig. 2E). For the biased adaptation condition, 8 directions were presented with an equal probability before adaptation. During the biased adaptation, the adapting grating was 3 times more likely to be presented than any other grating, and was presented for 12 repetitions for adapting grating and 3 repetitions for each non-adapting grating in a trial, with 5–20 trials presented for each subject (Fig. 5B). Moving random dots stimuli for optical imaging were identical to

those described in the top-up adaptation with drifting gratings. For adaptation protocol with flashing gratings stimuli, flashing gratings were randomly presented at 4 orientations (45-deg increments). The time course of stimuli was in accordance with that used in drifting grating stimuli.

The analyses of evoked potential signal.

Visually evoked potentials were amplified, band-pass filtered (0.3–300 Hz) and digitized at 10 kHz with the same system in single-unit recordings. Raw visually evoked potential signals were filtered by low-pass filtering using a 4th-order Butterworth filter (cut-off frequency of 35 Hz), and the signals that included at least 10 repetitions were averaged [2]. Two peaks were observed in visually evoked potentials around 40–75 ms and 80–160 ms, respectively. The mean value of the baseline signals (0–200 ms prior to the stimulus onset) was defined as the baseline for quantitative comparison.

References

1. Engel, S. a. Adaptation of oriented and unoriented color-selective neurons in human visual areas. *Neuron* **45,** 613–23 (2005).
2. Di Russo, F. *et al.* Identification of the neural sources of the pattern-reversal VEP. *Neuroimage* **24,** 874–886 (2005).

Table and Figures

Relative Ori. to Peak Response Ori. (deg)		0	±15	±30	±45	±60	±75	±90
**Response Ratio**	Area17	0.84±0.05	0.91±0.06	1.13±0.16	1.15±0.18	1.28±0.17	1.39±0.20	1.84±0.28
**(mean±SEM)**	Area 21a	0.58±0.06**	0.68±0.11*	0.93±0.14	1.53±0.27	1.36±0.24	1.31±0.22	1.57±0.34

Supplementary Table 1. Adaptation effects at different relative orientations to the adapting orientation.

*p<0.05, **p<0.01, t-test; n=51 for area 17, and n=39 for area 21a.

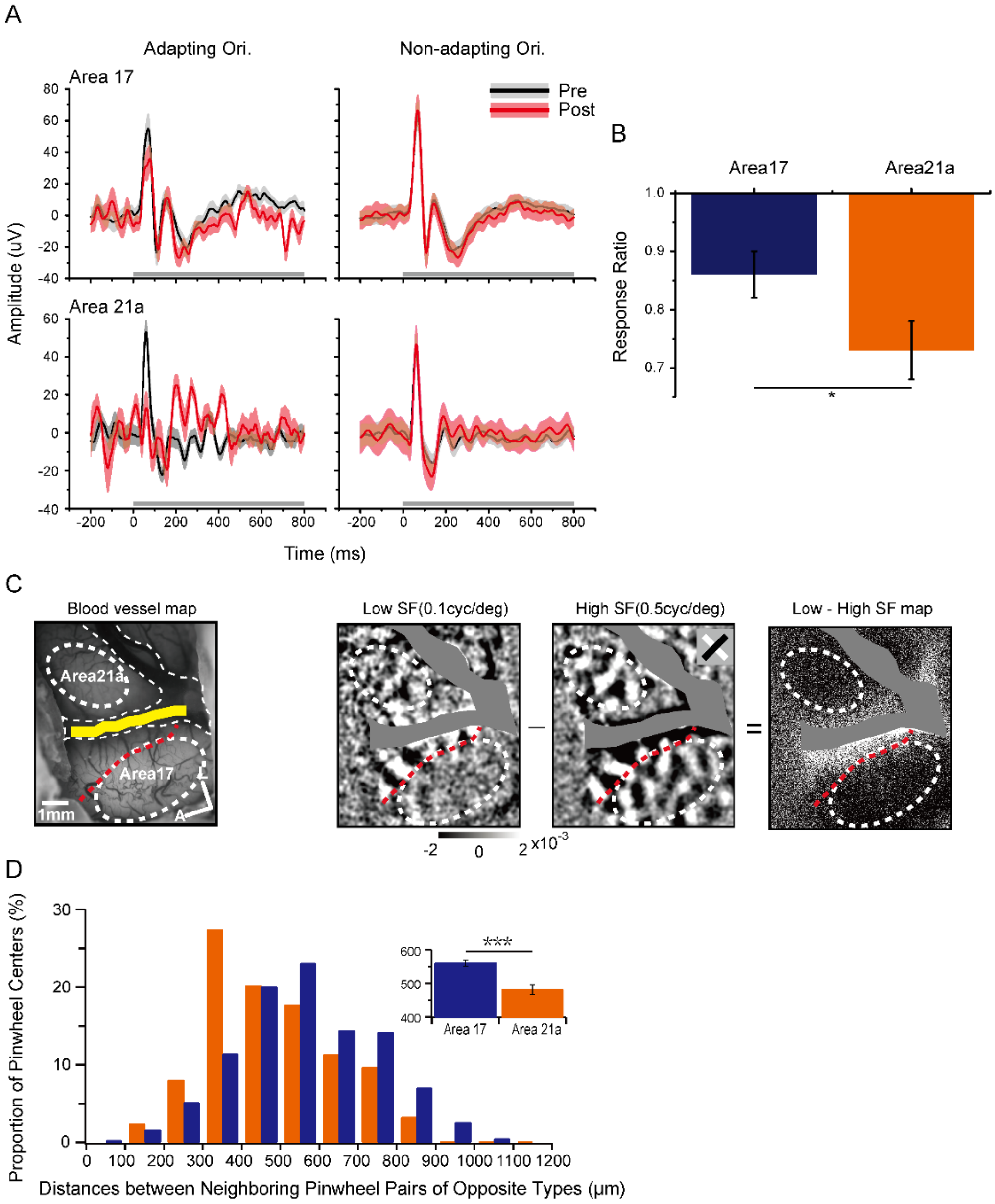

We modified the captions of Supplementary Figure 1 as the following:

Supplementary Figure 1. Top-up orientation adaptation in areas 17 and 21a measured by visually evoked potentials and delineation of areas 17 and 21a. **A**, Examples of visually evoked potential evoked by adapting orientation (left) and non-adapting orientation (right) before (black lines) and after adaptation (red lines) in area

17 (top) and area 21a (bottom). Stimulus duration is indicated by gray bars. Shadows indicate SEM (n=20 repeats). **B**, A statistical comparison of the visually evoked potential response ratios in area 17 (blue bar) and area 21a (orange bar) (n=23, 26 recording sites, respectively, p=0.02, t-test). **C**, The first column, blood vessel map (same as Fig.2A), red dash line indicates 17/18 border. ROI (elliptical area) for calculating the strength of global signals in area 17 and area 21a. Yellow bar indicates sulcus lateralis (separation of area 21a and area 17/18) with a large blood vessel. The second and third column, orientation differential map in areas 17, 18 and 21a with low SF grating (second, 0.1 cyc/deg) and high SF grating (third, 0.5 cyc/deg). The fourth column, low—high spatial frequency map. Dark and light indicate the preference for high and low spatial frequency, respectively. This spatial frequency helped to differentiate the 17/18 border (red dash line). **D**, Distribution histograms (pre-adaptation conditions) of the distance between pairs of neighboring pinwheel centers with opposite types (clockwise and counterclockwise) in areas 21a (orange bar) and 17 (blue bar). On average, the mean pinwheel pairs distance in areas 17 was greater than that in area 21a (area 17: 559.66±8.71 um, n=430; area 21a: 480.58±14.56 um, n=124, p<0.0001, t-test). Error bars indicate SEM. *p<0.05. ***p<0.0001.

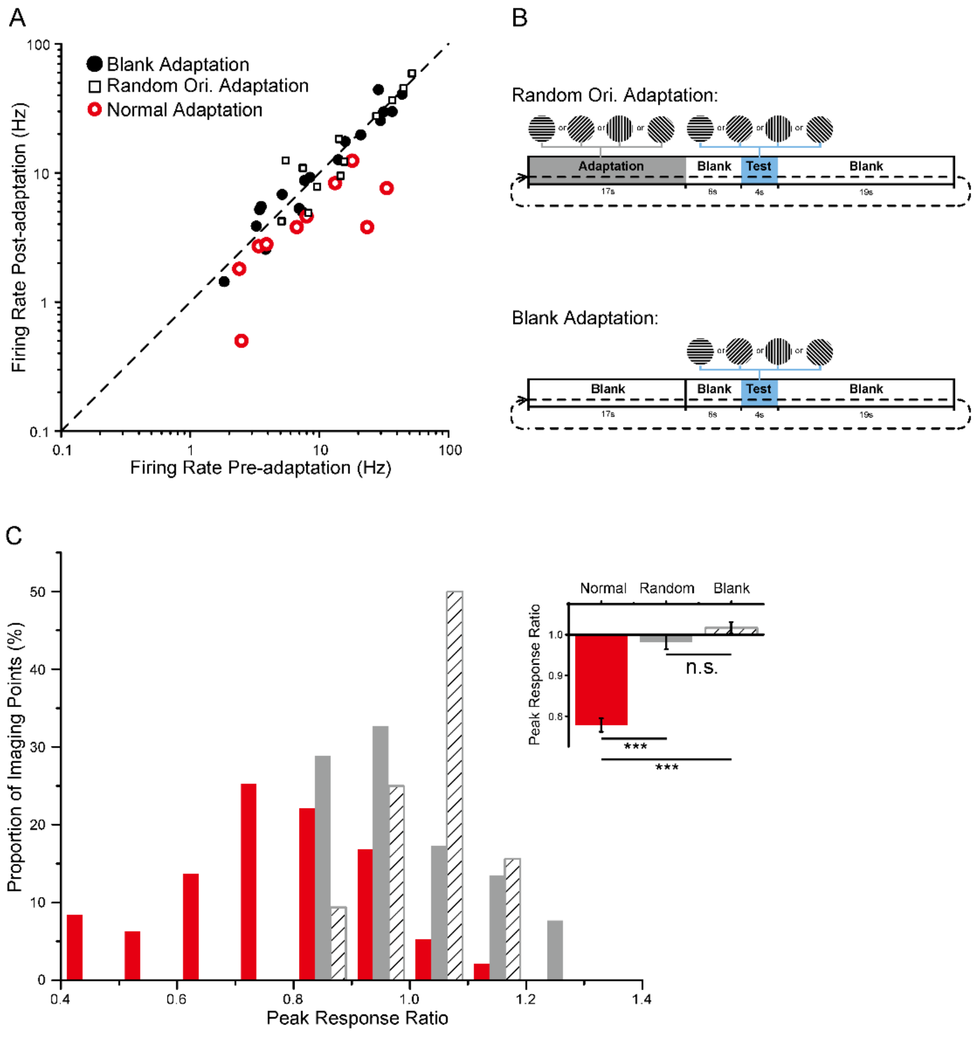

Supplementary Figure 2. Top-up adaptation effects of area 17 evoked by different adaptation protocols. **A**, A comparison of the peak responses before and after adaptation in area 17 measured by single-unit recordings, a blank adaptation protocol (black circles) , a random-orientation adaptation protocol (black boxes) and a long-term top-up adaptation protocol (red circles) were applied. **B,** Control top-up adaptation protocols with 4 randomly presented orientations adapting stimuli (top) or blank stimulus (bottom) in area 17. **C,** The distribution histograms of the peak response ratios in normal adaptation (red bars), blank (forward-slash bar) and random

adaptation (gray bar), measured by intrinsic signal optical imaging. Comparisons of the peak response ratios with these top-up adaptation protocols (normal vs blank, $p<0.0001$; normal vs random, $p<0.0001$; blank vs random, $p=0.55$, one-way ANOVA). Error bars indicate SEM. ***$p<0.0001$.

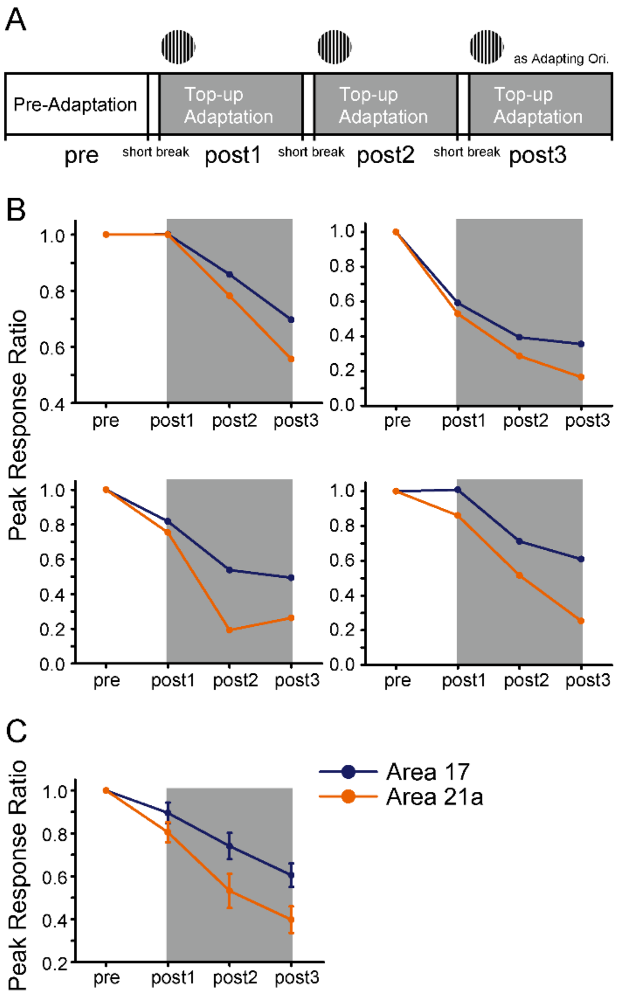

Supplementary Figure 3. Temporally dynamic procedure of top-up adaptation measured by optical imaging. **A**, Sequential top-up adaptation protocol. Each individual adaptation session (post 1or post 2 or post 3) lasted for 37 min, and a short break about 2 minutes was inserted in between. **B-C**, Four typical cases (B) and the average of 9 cats (C). Peak response ratio curves during sequential top-up adaptations in area 17 (blue) and area 21a (orange). Error bars indicate SEM.

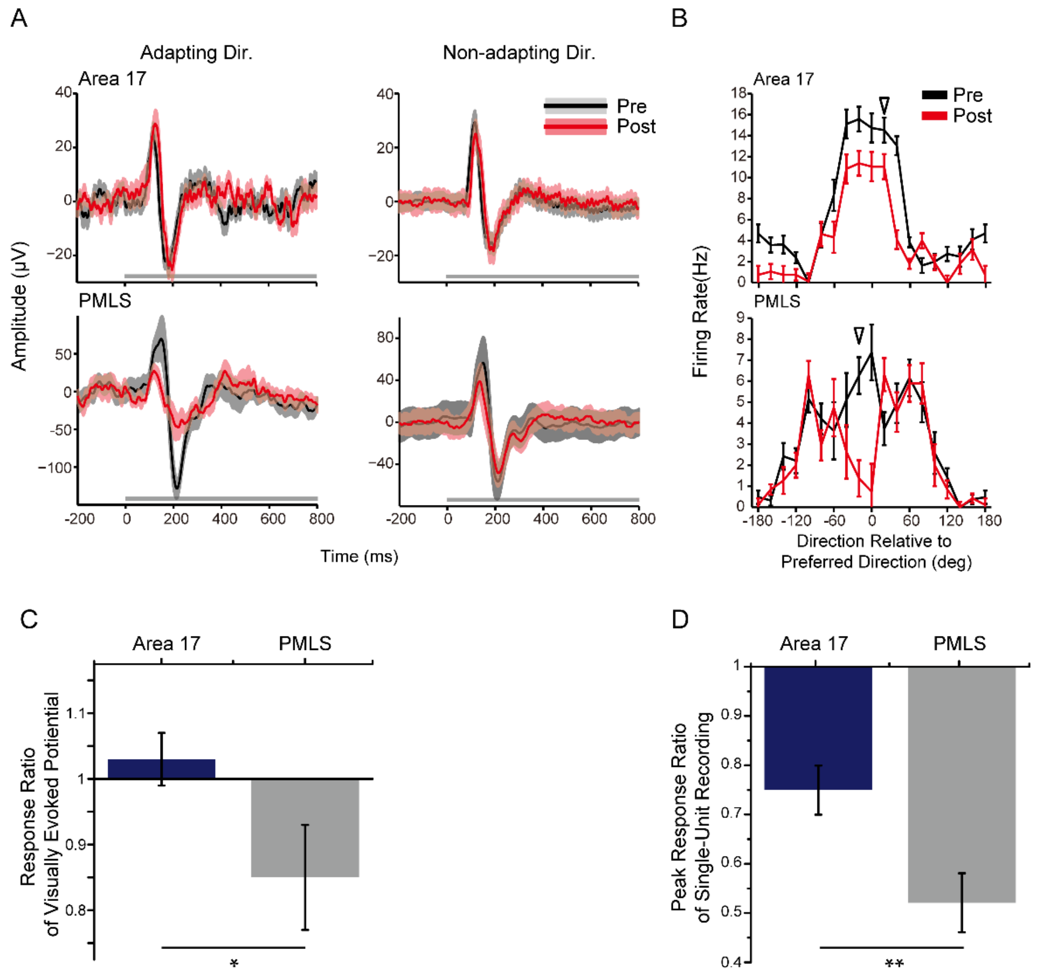

Supplementary Figure 4. The inherited direction adaptation in PMLS. **A**, An example showing visually evoked potential response to adapting direction (left) and non-adapting direction (right) before (black lines) and after top-up direction adaptation (red lines) in area 17 (top) and PMLS (bottom) by moving random dots. Error shadows indicate SEM (n=20 repeats). **B**, Direction-tuning curves of example neurons in area 17 (top, the same neuron in Fig. 10A) and PMLS (bottom) before (black lines) and after (red lines) top-up direction adaptation measured by single-unit recordings. Arrowhead indicates the direction of the adaptor. Error bars indicate SEM

(n=10 repeats). **C**, The statistics of response ratios in area 17 (n=21, blue bar) and PMLS (n=12, gray bar) after top-up direction adaptation, measured by visually evoked potentials (p=0.016, t-test). Error bars indicate SEM. **D**, The statistics of peak response ratios in area 17 (n=11, blue bar) and PMLS (n=17, gray bar) after top-up direction adaptation, measured by single-unit recordings (p=0.005, t-test). Error bars indicate SEM. **p<0.001.
